# *Boehmeria nivea* (L.) Gaud. *ameliorate oxidative stress-mediated inflammatory Responses and apoptosis in LPS/CSC-induced chronic obstructive pulmonary disease mouse model*

**DOI:** 10.3389/fphar.2025.1710694

**Published:** 2026-01-28

**Authors:** Ba-Wool Lee, Ji-Hye Ha, Da-Hye Yi, Ju-Hong Kim, Seong-Hun Jeong, Ju Hwan Jeong, Kyungsook Jung, Hyung Jae Jeong, Ji-Young Park, Woo Sik Kim, Young-Bae Ryu, Hyung-Jun Kwon, Jong-Choon Kim, In-Sik Shin, In-Chul Lee

**Affiliations:** 1 Functional Biomaterial Research Center, Korea Research Institute of Bioscience and Biotechnology, Jeongeup-si, Jeollabuk-do, Republic of Korea; 2 College of Veterinary Medicine, Chonnam National University, Gwangju, Republic of Korea; 3 College of Veterinary Medicine, Chungnam National University, Daejeon, Republic of Korea

**Keywords:** chronic obstructive pulmonary disease, Boehmeria nivea (L.) Gaud., oxidative stress, thioredoxin-interacting protein, NLRP3 inflammasome, apoptosis

## Abstract

**Introduction:**

*Boehmeria nivea* (L.) Gaud. has traditionally been regarded as a medicinal food with applications in various inflammatory disorders. However, its role in chronic obstructive pulmonary disease (COPD) has not yet been clarified.

**Methods:**

In this study, the preventive efficacy of the ethyl acetate fraction of *B. nivea* (L.) Gaud. leaves (EA-BN) was evaluated in a COPD model established by intratracheal instillation of lipopolysaccharide (LPS; 0.5 mg/kg body weight) and cigarette smoke condensate (CSC; 12.5 mg/kg body weight) in male C57BL/6N mice. The experimental groups received dexamethasone (3 mg/kg) as a positive control or EA-BN at doses of 100 and 200 mg/kg.

**Results:**

EA-BN administration significantly reduced T helper 1 cytokine levels and decreased macrophage and neutrophil counts in bronchoalveolar lavage fluid. Histological analyses revealed that EA-BN mitigated alveolar destruction and inflammatory infiltration, whereas pulmonary function tests demonstrated improvements in the FEV0.1/FVC ratio and lung elastance in the LPS/CSC-induced COPD. Additionally, EA-BN alleviated oxidative stress by promoting the nuclear translocation of Nrf2 and enhancing the expression of its downstream targets, HO-1 and NQO1, leading to a reduction in reactive oxygen species and nitric oxide production. EA-BN downregulated thioredoxin-interacting protein and NLRP3 inflammasome activation, thereby suppressing caspase-1 and IL-1β expression, whereas also attenuating apoptosis by modulating the Bax/Bcl-2/caspase-3 pathway.

**Discussion:**

Collectively, these findings suggest that EA-BN possesses antioxidant, anti-inflammatory, and anti-apoptotic properties, supporting its potential as a preventive agent against COPD.

## 1 Introduction

Chronic obstructive pulmonary disease (COPD) is a refractory respiratory disorder characterized by persistent respiratory symptoms and airflow limitation caused by airway and/or alveolar abnormalities (Katamesh et al., 2025; Macleod et al., 2021). This phenomenon is associated with multiple factors, including oxidative stress, impaired immune repair, and chronic inflammation (Macleod et al., 2021; Zhang et al., 2020). Cigarette smoke is a major risk factor for COPD and contains approximately 4,500 different chemicals, such as oxidants, tar, carcinogens, and toxic gases (Li et al., 2018; Pinto et al., 2017). Prolonged exposure to cigarette smoke activates inflammatory cells such as macrophages and neutrophils, which secrete ROS, Th1 cytokines, and proteolytic enzymes, thereby causing alveolar destruction and extracellular matrix degradation (Hakansson et al., 2012; Ma et al., 2022; Wu et al., 2022). Therefore, a multitarget therapeutic strategy that concurrently addresses oxidative stress, inflammation, and fibrosis could be an effective approach for regulating COPD. This approach may simplify the complex pathogenesis of COPD, potentially leading to improved patient outcomes.

ROS-induced oxidative stress is a critical driver of COPD progression and contributes to extracellular matrix remodeling, alveolar cell death, and bronchiolar obstruction, which collectively impair pulmonary function (Tabeshpour et al., 2024; Yoshida et al., 2019). Persistent oxidative stress amplifies inflammatory responses and disrupts tissue homeostasis, exacerbating the severity of COPD. Nuclear factor erythroid 2-related factor 2 (Nrf-2) plays a pivotal role in regulation of oxidative stress by neutralizing ROS and protecting cells from oxidative damage (Wang et al., 2019). In response to oxidative stress, Nrf-2 dissociates from the Kelch-like ECH-associated protein 1 (Keap-1) and translocates to the nucleus, where it binds to antioxidant response elements (AREs). This interaction triggers transcriptional activation of various antioxidant and cytoprotective enzymes, including NAD(P)H quinone dehydrogenase 1 (NQO1) and heme oxygenase 1 (HO-1), which are essential for mitigating oxidative damage (Xu et al., 2024; Yoshida et al., 2019). As a key antioxidant defense system, the Nrf-2/HO-1/NQO1 signaling pathway upregulation has emerged as a promising therapeutic target for slowing COPD progression and improving disease management.

A comprehensive understanding of lung injury requires the identification of molecular markers that reflect the interconnection between inflammation, oxidative stress, and apoptosis, which together drive COPD progression (Dou, et al., 2025; Zhou et al., 2024). The thioredoxin (TXN)/thioredoxin interacting protein (TXNIP) complex plays a key role in redox homeostasis and is associated with oxidative stress-related disorders, such as diabetes, autoimmune disorders, cancer, and apoptosis (Kansal et al., 2023; Zhou and Chng, 2013). Excessive ROS disrupts the TXN-TXNIP complex, leading to TXNIP-mediated activation of the NLRP3 inflammasome, which subsequently induces caspase-1 cleavage and maturation of IL-1β and IL-18, thereby exacerbating chronic inflammation in COPD (Kim J et al., 2024; Wang et al., 2025). Dissociated TXNIP also promotes apoptosis via the Bax/Bcl-2/caspase-3 pathway, in which an imbalance in the Bax/Bcl-2 ratio impairs mitochondrial integrity. This disruption triggers cytochrome c release, caspase-3 activation, and airway remodeling, resulting in lung destruction (Deng et al., 2017; Fan et al., 2020; Liu et al., 2023). Collectively, these findings suggest that targeting TXNIP-driven NLRP3 inflammasome activation and Bax/Bcl-2-mediated apoptosis, along with the assessment of oxidative and inflammatory biomarkers, represents a promising therapeutic strategy for counteracting oxidative stress-induced inflammation and lung injury in COPD.

*Boehmeria nivea* (L.) Gaud. (BN), a member of the Urticaceae family, is native to Southeast Asia and is commonly found in countries such as the Republic of Korea, China, the Philippines, and India, thriving in warm and humid climates (Wei et al., 2014; Lee et al., 2020). Traditionally, various parts of BN, including the roots, leaves, and flowers, have been utilized in folk medicine for their hemostatic, hepatoprotective, diuretic, antioxidant, and anti-inflammatory properties (Bae, 2000; Lin et al., 1998). Experimental evidence has highlighted its pharmacological properties, including reducing nitric oxide (NO) and ROS levels in lipopolysaccharide (LPS)-stimulated RAW264.7 cells and protecting glial cells exposed to hydrogen peroxide (Sung et al., 2013; Wang et al., 2015). In addition to cigarette smoke exposure, other contributing factors such as bacterial, fungal, and viral infections, as well as air pollutants, are known to exacerbate COPD symptoms (Oladipo et al., 2025; Park et al., 2022). Therefore, in this study, LPS was employed in combination with cigarette smoke condensate (CSC) to better mimic the complex inflammatory environment observed in COPD. However, the protective effects of the ethyl acetate extract of *Boehmeria nivea* (L.) Gaud. (EA-BN) in COPD have not yet been explored. Therefore, we evaluated the protective effects of EA-BN in COPD using LPS- and CSC-exposed cells and mice. We aimed to explore EA-BN as a novel preventive agent for COPD and to elucidate its mechanisms of action by examining the expression of key proteins involved in inflammatory responses and oxidative stress, both of which are central to COPD pathogenesis.

## 2 Materials and methods

### 2.1 Plant material and UPLC-Q-TOF-MS analysis

*Boehmeria nivea* (L.) Gaud. leaves were collected from Naejangsan Mountain (Jeongeup-si, Republic of Korea), shade-dried, and powdered. The powdered material (100 g) was extracted with 1 L of ethyl acetate by ultrasonic-assisted extraction (40 kHz, 30 cycles, 15 min each). The extract was then filtered, concentrated under reduced pressure, and yielded 3.388 g of the ethyl acetate fraction of *B. nivea* (L.) Gaud. (EA-BN). For chemical profiling, EA-BN was analyzed using UPLC coupled with a Q-TOF mass spectrometer on a BEH C18 column (2.1 × 100 mm, 1.7 μm). The mobile phase consisted of 0.1% formic acid in water (A) and acetonitrile (B), with a gradient from 5% to 100% B over 20 min. Mass spectrometric analysis was performed in positive ion mode (*m/z* 100–1500, scan time 0.2 s) with a desolvation temperature of 350 °C, nitrogen flow of 800 L/h, source temperature of 110 °C, and cone voltage of 40 V. Leucine-enkephalin ((M + H)^+^ m/z 556.2615) was used as the lock mass, and data acquisition was conducted using UNIFI software (Waters Corp., USA).

### 2.2 Chemicals and materials

The RAW264.7 cell line was obtained from the Korean Collection for Type Culture (Seoul, Republic of Korea). LPS (*E. coli*; 0111: B4, Cat. Num. L2630), tribromoethanol (Avertin), and dexamethasone (DEX) were sourced from Sigma-Aldrich (MO, USA). Cigarette smoke condensate (CSC) was obtained from the Korean Institute of Toxicology (Jeongeup-si, Republic of Korea). Various assay kits were utilized following the manufacturer’s protocols, including the ROS detection assay kit (DCF-DA; CellRox® green reagent; Thermo Scientific, MA, USA), EZ-Glutathione Assay Kit, EZ-Catalase Kit, EZ-Lipid Peroxidation (TBARS) Assay Kit (DoGen, Seoul, Republic of Korea), and the Nitric Oxide Plus Kit (iNtRON Biotechnology, Republic of Korea). Additionally, ELISA kits for TNF-α, IL-6, and IL-1β (R&D System, MN, USA) and the Diff-Quik® Stain kit (IMEB, CA, USA) were employed in accordance with the respective manufacturer’s instructions.

### 2.3 COPD animal model and experimental procedure

C57BL/6N male mice were obtained from Orient Bio (Republic of Korea) and maintained in accordance with the guidelines of the Institutional Animal Care and Use Committee (IACUC) of KRIBB (Approval Num.: KRIBB-AEC-23317). To establish a COPD mouse model, animals were exposed to LPS and cigarette smoke condensate (CSC). Specific pathogen-free male C57BL/6N mice were randomly divided into the following five groups (*n* = 7 per group).

- Normal control (NC) group: treated with vehicle (2% DMSO) from days 1 to day 14 and phosphate-buffered saline (PBS) intranasal instillation.
- COPD group: treated with vehicle (2% DMSO) from day 1 to days 14 and LPS/CSC intranasal instillation.
- DEX group: treated with dexamethasone (3 mg/kg) from day 1 to days 14 and LPS/CSC intranasal instillation.
- EA-BN-L group: treated with EA-BN-L (100 mg/kg; 2% DMSO) from day 1 to days 14 and LPS/CSC intranasal instillation.
- EA-BN-H group: treated with EA-BN-H (200 mg/kg; 2% DMSO) from day 1 to days 14 and LPS/CSC intranasal instillation.

The LPS/CSC-induced COPD model was developed following the procedures outlined by Kwak and Lim (2014) and Park et al. (2022). LPS (0.5 mg/kg) and CSC (12.5 mg/kg) were intranasally instilled under light anesthesia on days 1, 6, and 13. All animals received their designated oral treatments once daily from day 1 to day 14, and were euthanized for sample collection on day 15. Dexamethasone (3 mg/kg) served as the positive control, as previously described by Kim W et al. (2024).

### 2.4 Bronchoalveolar lavage fluid (BALF) analysis

For BALF collection, mice were tracheostomized under anesthesia, followed by endotracheal tube insertion. PBS (0.7 mL) was flushed into the lungs and withdrawn, and this process was repeated once, yielding a total BALF volume of 1.4 mL. BALF inflammatory cell counts were analyzed following the protocol described by Park et al. (2022). For cell count analysis, the collected BALF samples were centrifuged at 800 rpm for 10 min, after which the cell pellets were re-suspended in 1.4 mL of PBS and applied to slides using a Cytospin four centrifuge (Thermo Scientific) at 800 rpm for 5 min at 20 °C. To evaluate total cell count, slides were stained using the Diff-Quik® reagent, and the number of macrophages, lymphocytes, neutrophils, and total cells was recorded. The supernatants were stored at −70 °C for subsequent cytokine analysis.

### 2.5 Measurement of cytokine levels and oxidative stress markers

Th-1 cytokine levels in culture supernatants from BALF fluids and LPS/CSC-stimulated RAW264.7 cells were measured using competitive ELISA kits (R&D Systems) and analyzed with a plate reader set at 450 nm (Bio-Rad Laboratories, USA). RAW264.7 cells were cultured in 6-well plates and pretreated with EA-BN (50, 100, and 200 μg/mL) for 1 h prior to stimulation with LPS/CSC (0.5/2.5 μg/mL). Following LPS/CSC treatment, the cells were incubated for an additional 1 h and then harvested for subsequent analyses. The lung tissues were homogenized in PBS and centrifuged at 14,000 rpm for 15 min at 4 °C to collect the supernatants. ROS and nitric oxide (NO) levels were assessed using the CellRox® Green Reagent (Thermo Scientific) and Nitric Oxide Plus Kit (iNtRON Biotechnology), respectively, following the instructions provided by the manufacturers. Additionally, the content of recued glutathione (GSH), thiobarbituric acid reactive substances (TBARS), and activity of catalase was assessed by using EZ-Glutathione assay kit, EZ-catalase kit, and EZ-lipid peroxidation (TBARS) assay kit (DoGen, Seoul, Republic of Korea) according to the provided instructions.

### 2.6 Pulmonary function analysis

To assess pulmonary function, mice were anesthetized with 2.5% avertin (Sigma-Aldrich) via intraperitoneal injection (0.02 mL/g body weight), followed by a tracheostomy. Lung function was measured using a FlexiVent system (SCIREQ Scientific Respiratory Equipment Inc., Canada). Lung volume was determined through deep inflations (6 s), while total lung elastance and tissue elastance were analyzed using the Snapshot and Quick Prime-3 techniques, respectively (Li et al., 2020). Additionally, forced expiratory volume at 0.1 s (FEV_0.1_) and forced vital capacity (FVC) were assessed through the Negative Pressure-Driven Forced Expiration (NPFE) maneuver. The FEV_0.1_/FVC ratio was calculated using FlexiVent software (Shalaby et al., 2010).

### 2.7 Histopathological analysis

The lung tissue samples were fixed in 10% phosphate-buffered formalin, embedded in paraffin wax, and sectioned into 4 μm slices. These tissue sections were then stained with hematoxylin and eosin (H&E; BBC Biochemical, USA). The stained specimens were examined under a light microscope (Leica Microsystems) to evaluate airway inflammation, alveolar morphology, mean alveolar number (MAN), and mean linear intercept (MLI). The severity of inflammation was graded on a scale of 0–4 (0 = none, 1 = minimal, 2 = mild, 3 = moderate, and, 4 = severe). For the quantification of MAN and MLI, the number of alveolar intercepts (NS) and alveoli (Na) within a defined field (S) were measured, and the indices were calculated as MLI = L/NS and MAN = Na/S. Statistical analysis was performed using mean values from eight randomly selected microscopic fields.

### 2.8 Immunoblotting

Lung tissue proteins were extracted using Protein Extraction with protease and phosphatase inhibitors (Thermo Scientific). Equal amounts of protein (30 μg) were separated on a 4%–12% SDS-PAGE gel and transferred to polyvinylidene fluoride membranes. Membranes were blocked to minimize nonspecific binding and then incubated overnight with primary antibodies, including caspase-1, IL-1β, NQO1, HO-1, and Nrf-2 (Abcam, Cambridge, UK); TXNIP (Novus Bio, Centennial, CO, USA); NLRP3, Bax, Bcl-2, caspase-3, and β-actin (Cell Signaling Technology); and α-tubulin and Lamin B (Thermo Scientific). After 24 h, membranes were washed with TBST and exposed to secondary antibodies (0.1 μg/mL, Cell Signaling Technology) for 1 h. Protein bands were visualized with an enhanced chemiluminescence detection kit (Thermo Scientific) and quantified using a chemiluminescence scanner (LI-COR Biosciences, Lincoln, NE, USA).

### 2.9 Cell culture and cytotoxicity

RAW264.7 cells were cultured in DMEM supplemented with 10% heat-inactivated fetal bovine serum and 1% antibiotics in a 5% CO_2_ incubator (37 °C). RAW264.7 cells (5 × 10^4^ cells/well) were grown in 96-well plates for 24 h. The cells were incubated with different concentrations of EA-BN (0, 12.5, 25, 50, 100, 150, and 200 μg/mL) for 24 h. The plate incubated with EZ-CyTox (DoGen) for 1 h and the absorbance was conducted at 450 nm.

### 2.10 Analysis of annexin-V/propidium iodide (AV/PI) dual staining in LPS/CSC-stimulated RAW264.7 cells

Cell apoptosis was assessed using the Annexin V-FITC apoptosis detection kit (Abcam, UK), following the manufacturer’s protocol. RAW264.7 cells (5 × 10^5^ cells per well) were seeded into 6-well plates and pre-treated with various concentrations of EA-BN for 1 h before being stimulated with LPS/CSC (0.5/2.5 μg/mL). After 24 h of incubation, the cells were harvested and resuspended in Annexin V-FITC binding buffer. Subsequently, they were incubated with Annexin V-FITC and propidium iodide (PI) in a light-protected environment for 15 min at room temperature. Apoptotic cell populations were analyzed using a flow cytometer (BD Biosciences, San Jose, CA, USA), and data processing was conducted with FlowJo software version 7.6 (FlowJo, TreeStar, USA).

### 2.11 Nrf2/HO-1/NQO1 levels in LPS/CSC-stimulated RAW264.7 cells

RAW264.7 cells were plated in 6-well plates and exposed to EA-BN (50, 100, and 200 μg/mL) for 1 h before the addition of LPS/CSC (0.5/2.5 μg/mL). Following a 2 h incubation period, the cells were washed with PBS and then were separated to nuclear and cytoplasmic proteins using NE-PER Nuclear and Cytoplasmic Extraction Reagents (Thermo Scientific) following the manufacturer’s instruction. The levels of Nrf-2/HO-1/NQO1 and β-actin expression were evaluated by following immunoblotting.

### 2.12 Regulation of TXNIP-mediated NLRP3 inflammasome and apoptosis pathway by TXNIP silencing in LPS/CSC-stimulated RAW264.7 cells

TXNIP-targeting siRNA (catalog no. 4390771) and non-targeting control siRNA (catalog no. 4390843) were obtained from Ambion (Waltham, MA, USA). Transfection into RAW264.7 cells was carried out using Lipofectamine™ RNAiMAX reagent (Invitrogen, Waltham, MA, USA) according to the manufacturer’s forward transfection protocol, with each siRNA applied at a final concentration of 20 nM. Following successful knockdown of endogenous TXNIP expression, the cells were pretreated with concentration of EA-BN (200 μg/mL) for 1 h, then challenged with LPS/CSC (0.5/2.5 μg/mL). After a 2 h incubation, the cells were collected for analysis and the levels of TXNIP/NLRP3 with Bax/Bcl-2/caspase-3 pathway were assessed by immunoblotting.

### 2.13 RNA sequencing (RNA-seq) analysis and real-time PCR in lungs

Total RNA was isolated from mouse lung tissues using TRIzol reagent, and its quality was verified before downstream processing. RNA integrity was examined with the Agilent TapeStation RNA ScreenTape system (5,067–5,576; Agilent Technologies). Only high-quality RNA samples, as determined by the manufacturer’s recommendations, were used for library construction. Sequencing libraries were prepared with the TruSeq RNA Sample Prep Kit (Illumina) and subjected to paired-end sequencing (2 × 100 bp) at Macrogen (Seoul, Republic of Korea). Raw sequencing output was demultiplexed using bcl2fastq software (v2.2), followed by quality control assessment with FastQC. Gene expression read counts obtained through HTSeq were normalized with DESeq2 (v1.40.2). Differential expression analysis was performed using the lfcShrink function, with DEGs defined as those meeting the criteria of p < 0.05 and |log2 fold change| ≥ 1. Functional enrichment and pathway mapping of the DEGs were conducted using Molecular function and the Kyoto Encyclopedia of Genes and Genomes (KEGG) enrichment analysis pathway Database (https://www.kegg.jp/kegg/pathway.html).

Total RNA (1 µg) was reverse-transcribed into complementary DNA (cDNA) using the iScript™ cDNA Synthesis Kit (Bio-Rad Laboratories). Quantitative real-time PCR (qRT-PCR) was subsequently performed with the SensiFAST™ SYBR No-ROX Kit (BioLine, Taunton, MA, USA) according to the manufacturer’s instructions. The primer sequences used in this study are listed in Table 1.

**TABLE 1:** Primer sequences of real-time PCR.

Genes (10 pmol/μL)	Forward primer (5’ - 3′)	Reverse primer (5’ - 3′)	Accession number (GenBank)
Btk	AGG​ACT​GCT​GTG​ATG​TTC​TGG​A	CTG​CTG​TTG​ATG​GTC​TTG​GTG​A	NM_013482.2
Trim30a	CCT​GCT​TCA​GGA​CAC​CAT​CTT	GCT​GAC​ATT​CAG​GTT​GCT​GTT	NM_009099.2
Eif2ak2	ACC​TGC​TGT​TCT​TCC​TGC​TCT​A	GAT​GAC​CTT​CTC​CAG​CAG​GTT​A	NM_011163.4
Myd88	GAG​CTG​CTC​TTC​TGC​TTC​TTG​A	CAG​GAT​GCT​GCT​GTT​GAT​GTT​G	NM_010851.4
Igtp	TTC​TAC​CGC​CTG​CTC​ATC​TTC​T	AGG​TCT​TGG​TGA​TGG​TGA​TGG​T	NM_018738.3
Gbp5	TGG​ACT​TCC​TGC​TGT​TCT​TCC​T	ACA​GGT​TGA​TGG​TGT​TGG​TGA​T	NM_172657.3
Irgm1	CTG​CTG​GAG​ATC​ATC​GAG​TTC​A	GGT​CTT​CTG​GTT​GAT​GGT​GTT​G	NM_008326.4
Irgm2	AGC​CTG​ACT​TCC​TGC​TGT​TCT​A	CTG​GTG​ATG​TTG​GTG​ATG​GTG​A	NM_001286414.1
ND1	CCC​TAA​AAC​CCG​CCA​CAT​CT	GAG​CGA​TGG​TGA​GAG​CTA​AGG​T	NC_005089.1 (mtDNA)
ND2	AGG​TTC​TTC​TGC​TCC​TGT​TGG​A	TCA​TGG​TAG​GAG​GTG​ATG​GTG​A	NC_005089.1 (mtDNA)
ND3	TTG​CTC​ATG​GTG​GTT​CTG​TTC​T	ACA​GGT​TGA​GGT​TGG​TGA​TGA​T	NC_005089.1 (mtDNA)
ND4	GCT​TCT​GCT​GTT​CTT​CCT​GTT​G	CAG​GAT​GTT​GGT​GAT​GGT​GTT​G	NC_005089.1 (mtDNA)
ND5	TGG​AGT​TCT​GCT​GTT​CTG​GTT​A	ACC​TGA​TGG​TGA​TGG​TGT​TGA​T	NC_005089.1 (mtDNA)
ND6	AGG​TGC​TGT​TCT​GCT​TCT​TGT​T	TCA​GGT​GAT​GGT​GAT​GGT​GTT​G	NC_005089.1 (mtDNA)

### 2.14 Statistical analyses

All data are expressed as the means ± standard deviation (SD). Statistical analysis carry out using analysis of variance (ANOVA), followed by a Tukey’s multiple comparison test. Differences were considered statistically significant at P values ≤0.05 and ≤0.01. All statistical procedures were conducted using GraphPad Software (CA, USA).

## 3 Results

### 3.1 Bioactive components in EA-BN extract

**FIGURE 1:**
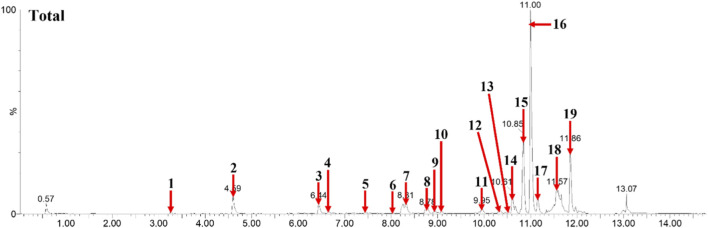
The metabolites contained in EA-BN were analyzed in positive mode using UPLC-QToF-MS.

The components of ethyl acetate of *B. nivea* (L.) Gaud. were evaluated using UPLC-Q-TOF MS. The linear relationships and detection limit of each component are shown in Figure 1, and results displayed that EA-BN contained ethyl gallate, dihydroferulic acid, caffeic acid, epicatechin, 12-oxophytodienoic acid, colnelenic acid, stearidonic acid, α-Linolenic acid, 4,8,12,15-octadecatetraenoic acid, 12,13-epoxy-9,15-octadecadienoic acid, arachidonic acid, octadeca-5,9,12-trienoic acid, 12-hydroxy-5Z,8Z,12Z-eicosatrienoic acid, two-o-protocatechuoylalphitolic acid, pheophorbide A, 1,3,6-tri-o-galloyl-beta-D-glucose, methyl phaeophorbide B, and fatty acids as presented in Table 2.

**TABLE 2:** Tentatively identification of major peaks detected in ethyl acetate extract of *Boehmeria nivea* (L.) Gaud. leaves.

No.	Retention time (min)	Identification	Exact mass (m/z)	Fragment ions (m/z)
1	3.26	Ethyl gallate	199.06	143, 127, 93
2	4.59	Dihydroferulic acid	197.12	179, 161, 151, 135, 119, 107
3	6.44	Caffeic acid	181.12	163, 145, 135, 107, 95
4	6.65	Epicatechin	291.19	273, 149, 135, 107, 91
5	7.45	12-Oxophytodienoic acid	293.21	275, 257, 221, 107, 81
6	8.04	Colnelenic acid	293.21	275, 257, 119, 93
7	8.31	Stearidonic acid	277.21	149, 135, 121
8	8.78	α-Linolenic acid	279.23	243, 173, 109, 95
9	8.93	4,8,12,15-Octadecatetraenoic acid	277.21	135, 107, 81
10	9.09	12,13-Epoxy-9,15-octadecadienoic acid	295.22	277, 151, 133
11	9.95	Arachidonic acid	305.25	161, 121, 107
12	10.31	Octadeca-5,9,12-trienoic acid	279.23	175, 135, 121, 95
13	10.53	12-Hydroxy-5Z,8Z,12Z-eicosatrienoic acid	323.26	277, 121
14	10.61	2-O-Protocatechuoylalphitolic acid	609.27	591
15	10.85			
16	11.00	Pheophorbide A	593.28	533
17	11.15			
18	11.57	1,3,6-Tri-O-galloyl-beta-D-glucose	637.30	619
19	11.86	Methyl phaeophorbide B	621.30	561

### 3.2 Effects of EA-BN on Th-1 cytokine levels and inflammatory cell counts in BALF of the LPS/CSC-induced COPD model

**FIGURE 2:**
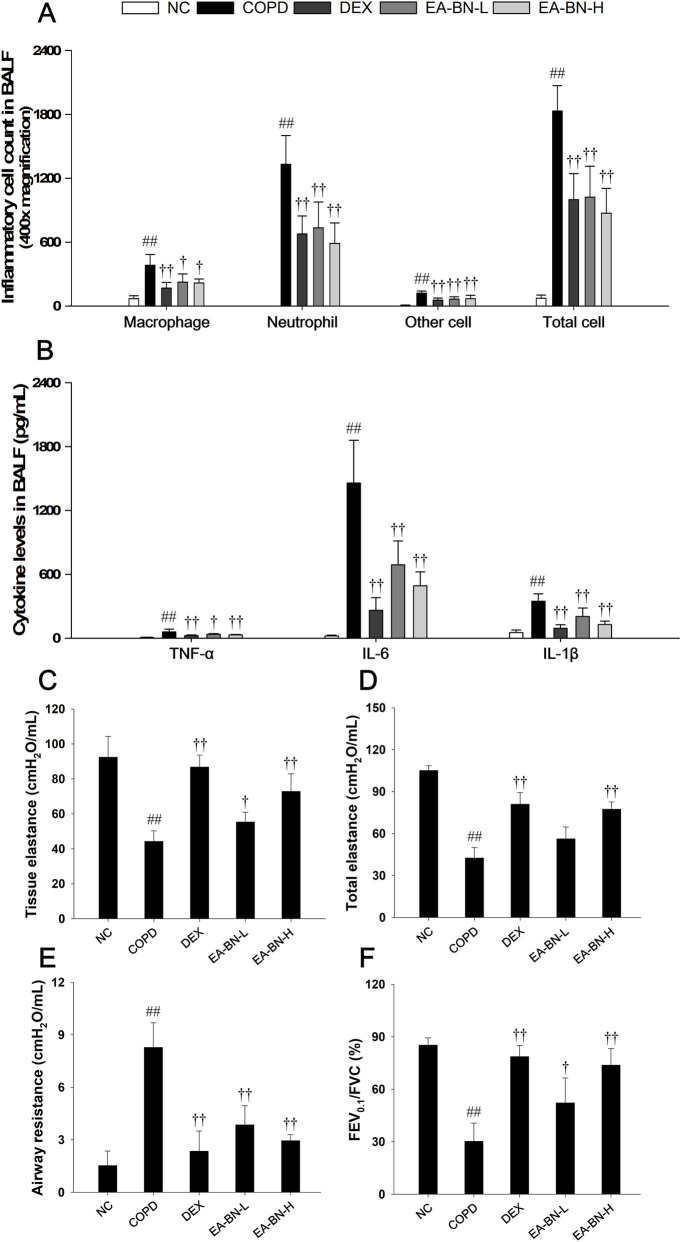
Effects of EA-BN on inflammatory cell counts, Th-1 cytokines, and pulmonary function in LPS/CSC-induced COPD model. **(A)** Inflammatory cells were visualized using Diff-Quik staining. **(B)** Th-1 cytokine concentrations (TNF-α, IL-1β, and IL-6) in BALF were quantified by ELISA. **(C)** Tissue elastance was assessed with the Quick Prime-3 maneuver. **(D)** Total elastance and **(E)** airway resistance in the respiratory system were evaluated by the snapshot perturbation maneuver. **(F)** Pulmonary function parameters, including forced expiratory volume at 0.1 s (FEV_0.1_), forced vital capacity (FVC), and the FEV_0.1_/FVC ratio, were determined in mice using the negative pressure-driven forced expiration technique. NC: normal control mice; COPD: LPS/CSC-induced mice; DEX: dexamethasone (3 mg/kg) + COPD; EA-BN-L or -H: EA-BN (100 or 200 mg/kg) + COPD. The values are expressed as the means ± SD (*n* = 7/group). ^##^ *P* < 0.01, significantly different from NC group; ^†,††^ *P* < 0.05, *P* < 0.01, significantly different from COPD.

Mice exposed to LPS/CSC exhibited a significant increase in inflammatory cell counts compared to the normal control group (Figure 2A). However, administration of EA-BN led to a notable reduction in inflammatory cell infiltration in comparison to LPS/CSC-induced COPD mice. In addition, the levels of IL-1β, IL-6, and TNF-α were markedly elevated in the BALF of LPS/CSC-induced COPD mice compared to non-treated mice (Figure 2B). In contrast, EA-BN treatment resulted in a significant, dose-dependent suppression of Th-1 cytokine levels when compared to the LPS/CSC-induced COPD group.

### 3.3 Effects of EA-BN on pulmonary function of the LPS/CSC-induced COPD model

Analysis of snapshot and Quick Prime-3 perturbation in LPS/CSC-induced COPD mice demonstrated a significant reduction in both total elastance (Figure 2C) and tissue elastance (Figure 2D) compared to the normal control group. Additionally, mice with LPS/CSC-induced COPD exhibited notably increased airway resistance (Figure 2E) and a decreased FEV_0.1_/FVC ratio (Figure 2F), indicating impaired lung function. In contrast, EA-BN-treated mice showed a significant suppression of airway resistance and improvement in total and tissue elastance, along with a higher FEV_0.1_/FVC ratio, compared to the LPS/CSC-induced COPD.

### 3.4 Effects of EA-BN on alveolar destruction and inflammatory cell infiltration in the lung of the LPS/CSC-induced COPD model

**FIGURE 3:**
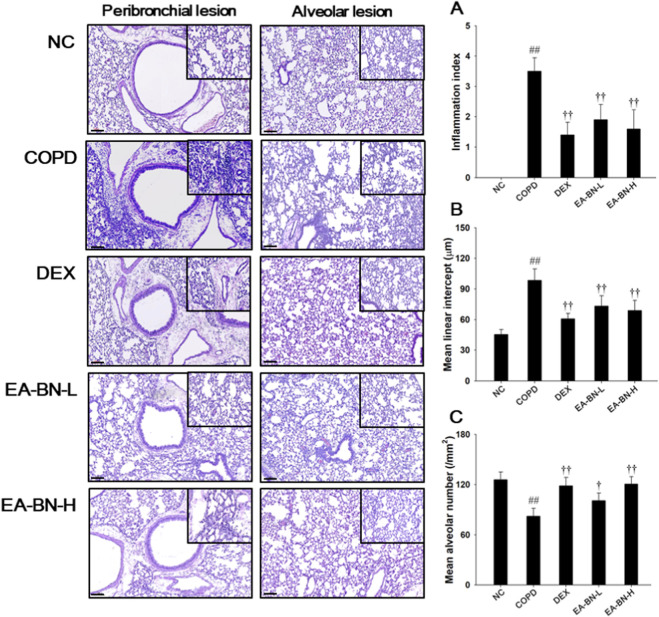
Effects of EA-BN on inflammatory cell infiltration and alveolar destruction in LPS/CSC-induced COPD model. A representative image of the airway and alveolar lesions in lung tissue stained with H&E. Quantification results of **(A)** inflammation index and alveolar destruction with airspace enlargement as **(B)** mean linear intercept and **(C)** mean alveolar number. Scale bars, 100 μm. NC: normal control mice; COPD: LPS/CSC-induced mice; DEX: dexamethasone (3 mg/kg) + COPD; EA-BN-L or -H: EA-BN (100 or 200 mg/kg) + COPD. The values are expressed as the means ± SD (*n* = 7/group). ^##^ *P* < 0.01, significantly different from NC group; ^†,††^ *P* < 0.05, *P* < 0.01, significantly different from COPD.

Lung tissue from LPS/CSC-induced COPD mice exhibited a significant increase in the inflammation index, indicating extensive inflammatory cell infiltration in the peribronchiolar and perivascular regions (Figure 3A), accompanied by airspace enlargement alveolar destruction (Figures 3B,C), compared with the normal control group. However, treatment with EA-BN markedly attenuated inflammatory cell accumulation in these regions and mitigated alveolar destruction and airspace enlargement, indicating a protective effect against LPS/CSC-induced lung inflammation and structural damage.

### 3.5 Effects of EA-BN on oxidative stress markers and Nrf-2 pathways in the lung tissue of the LPS/CSC-induced COPD model

**FIGURE 4:**
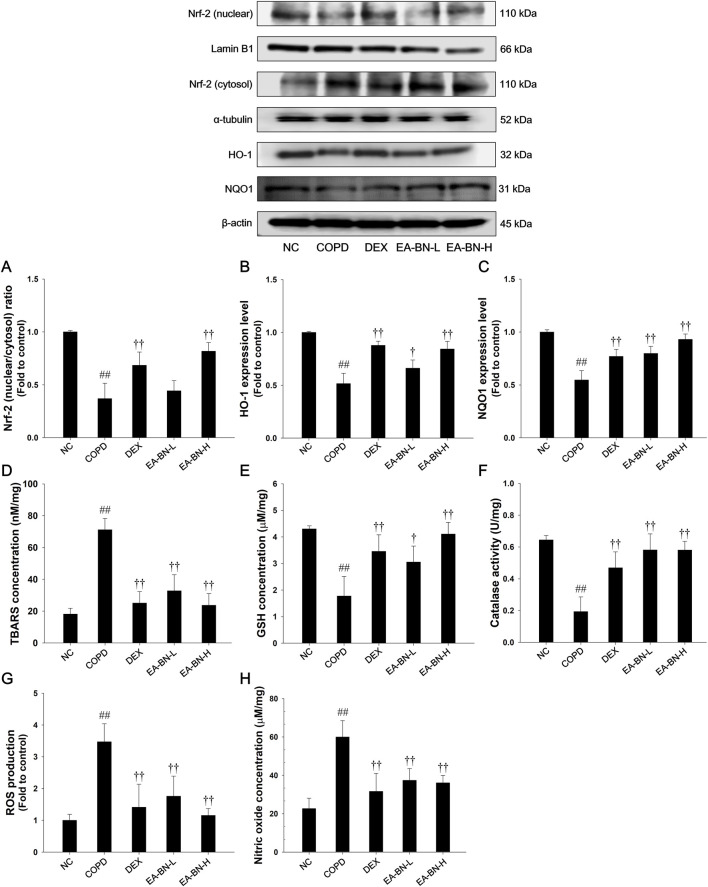
Effects of EA-BN on Nrf-2/HO-1/NQO1 expression and oxidative markers in lung of LPS/CSC-induced mice. Western blot analysis was performed to evaluate protein levels of **(A)** Nrf-2, **(B)** HO-1, and **(C)** NQO1 in lung tissue, with Lamin B1, α-tubulin, and β-actin used as loading controls. Oxidative stress indicators, including **(D)** TBARS, **(E)** GSH, **(F)** catalase activity, **(G)** ROS generation, and **(H)** NO concentration, were also quantified in lung samples. NC: normal control mice; COPD: LPS/CSC-induced mice; DEX: dexamethasone (3 mg/kg) + COPD; EA-BN-L or -H: EA-BN (100 or 200 mg/kg) + COPD. The values are expressed as the means ± SD (*n* = 7/group). ^##^ *P* < 0.01, significantly different from NC group; ^†,††^P < 0.05, P < 0.01, significantly different from COPD.

Mice with LPS/CSC-induced COPD exhibited a notable decrease in Nrf-2 nuclear translocation along with downregulated expression of HO-1 and NQO1 in lung tissues compared to the normal control group (Figures 4A–C). Additionally, these mice displayed a significant increase in TBARS, NO, and ROS levels, while showing a decline in catalase activity and GSH content (Figures 4D–H), indicating heightened oxidative stress. In contrast, EA-BN treatment significantly upregulated Nrf-2, HO-1, and NQO1 expression, suggesting enhanced antioxidant defense mechanisms. Furthermore, EA-BN administration effectively reduced TBARS, NO, and ROS levels, while enhancing catalase activity and increasing GSH content, compared to LPS/CSC-induced COPD mice.

### 3.6 Effects of EA-BN on TXNIP/NLRP3 inflammasome and apoptotic signaling pathway in the lung tissue of the LPS/CSC-induced COPD model

**FIGURE 5:**
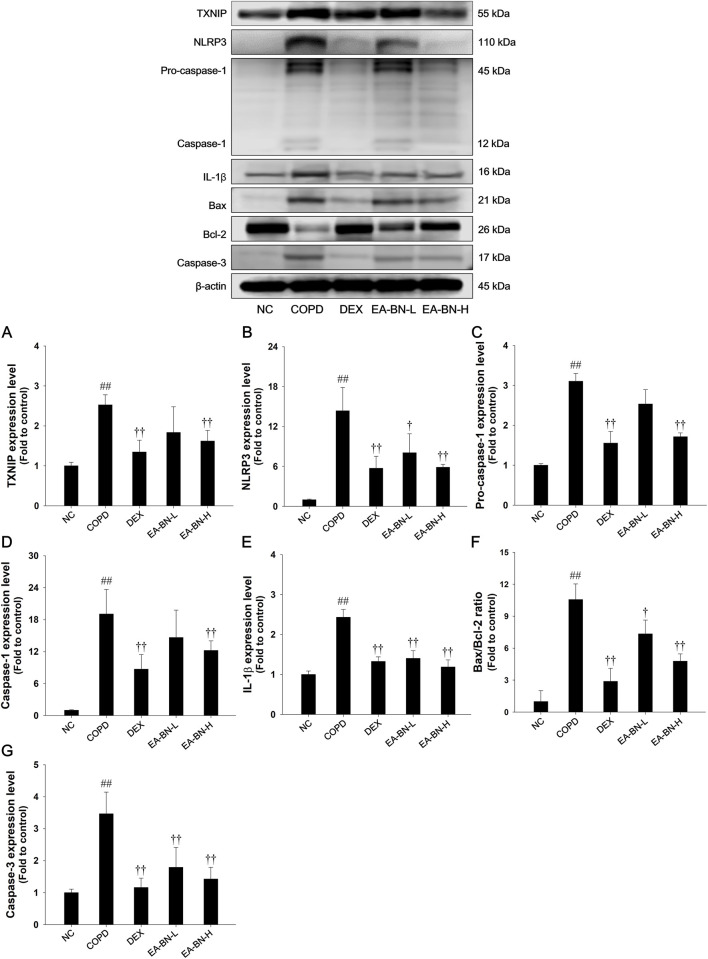
Effects of EA-BN on TXNIP/NLRP3 inflammasome and Bax/Bcl-2/caspase-3 signaling pathway in lung of LPS/CSC-induced mice. The protein levels of **(A)** TXNIP, **(B)** NLRP3, **(C)** pro-caspase-1, **(D)** caspase-1, **(E)** IL-1β, **(F)** Bax/Bcl-2 ratio, **(G)** caspase-3 were determined by western blot analysis in the lung tissues. β-actin was used to confirm equal protein loading. NC: normal control mice; COPD: LPS/CSC-induced mice; DEX: dexamethasone (3 mg/kg) + COPD; EA-BN-L or H: EA-BN (100 or 200 mg/kg) + COPD. The values are expressed as the means ± SD (*n* = 7/group). ^##^ *P* < 0.01, significantly different from NC group; ^†,^^††^ *P* < 0.05, P < 0.01, significantly different from COPD.

In the LPS/CSC-induced COPD mouse model, there was a significant upregulation of TXNIP/NLRP3 expression, accompanied by increased levels of the active forms of caspase-1 and IL-1β in lung tissues (Figures 5A–D). Additionally, these mice exhibited a notable increase in the Bax/Bcl-2 ratio, indicating a shift toward pro-apoptotic signaling, along with elevated expression of cleaved caspase-3 (Figures 5F,G). In contrast, EA-BN treatment significantly downregulated the expression of TXNIP and NLRP3, and effectively suppressed the activation of caspase-1 and IL-1β (Figures 5A–D). Moreover, EA-BN administration led to a reduction in the Bax/Bcl-2 ratio and decreased caspase-3 expression, suggesting that EA-BN alleviates apoptotic cell death in the LPS/CSC-induced COPD model (Figures 5F,G).

### 3.7 Effects of EA-BN on Th-1 cytokine in LPS/CSC-stimulated RAW264.7 cells

**FIGURE 6:**
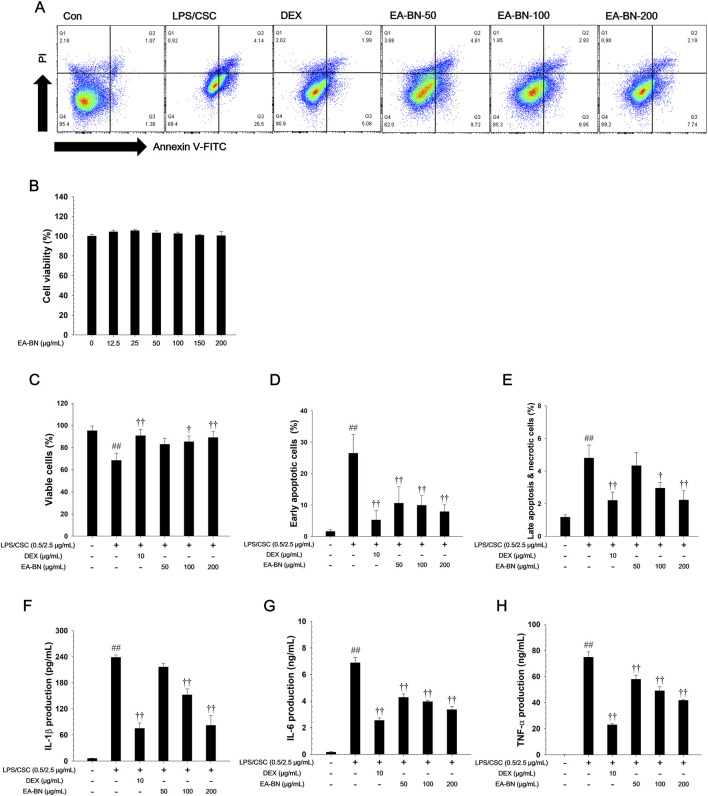
Effects of EA-BN on Th-1 cytokine production and apoptosis in LPS/CSC-stimulated RAW264.7 cells. **(A)** Representative flow cytometry plots showing apoptosis following EA-BN treatment in LPS/CSC-stimulated RAW264.7 cells. **(B)** Cell viability was determined by WST-1 assay after treatment with EA-BN (12.5, 25, 50, 100, and 200 μg/mL) for 24 h. Quantification of apoptotic responses showing **(C)** viable cells, **(D)** early apoptotic cells, and **(E)** late apoptotic/necrotic cells based on Annexin V-FITC/PI double staining. RAW264.7 cells were pretreated with EA-BN (50, 100, and 200 μg/mL) for 1 h, followed by exposure to LPS/CSC (0.5/2.5 μg/mL) for 24 h. The levels of Th-1 cytokines, **(F)** IL-1β, **(G)** IL-6, and **(H)** TNF-α, were quantified in culture supernatants using ELISA. Con: Non-stimulated cells; LPS/CSC: LPS/CSC-stimulated RAW264.7 cells; DEX: dexamethasone (10 μg/mL) + LPS/CSC-stimulated RAW264.7 cells; EA-BN: EA-BN (50, 100 and 200 μg/mL) + LPS/CSC-stimulated RAW264.7 cells. The values are expressed as the means ± SD (*n* = 3). ^##^ *P* < 0.01, significantly different from control; ^††^ *P* < 0.01, significantly different from LPS/CSC-stimulated RAW264.7 cells.

Different EA-BN concentrations were used in this experiment (Figure 6A). Annexin V-FITC/PI staining was performed to assess cell apoptosis and viability (Figures 6B–D). RAW 264.7 cells exposed to LPS/CSC showed a pronounced increase in Annexin V-positive apoptotic cells, along with a reduction in viable cell populations. Conversely, treatment with EA-BN significantly decreased apoptotic cell percentages while increasing the proportion of live cells in a dose-dependent fashion. LPS/CSC (0.5/2.5 μg/mL) treatment markedly increased the levels of Th-1 cytokines (Figures 6E–G). In contrast, EA-BN-treated cells showed lower level of Th-1 cytokines than LPS/CSC-stimulated cells, in a concentration-dependent manner. As shown in Figure 6A, different concentrations of EA-BN were applied to evaluate its immunomodulatory and cytoprotective effects. LPS/CSC (0.5/2.5 μg/mL) stimulation significantly increased the expression of Th-1 cytokines such as TNF-α, IL-6, and IL-1β (Figures 2B–D). However, EA-BN treatment markedly reduced cytokine levels in a concentration-dependent manner.

### 3.8 Effects of EA-BN on Nrf-2 pathways and oxidative stress makers production in LPS/CSC-stimulated RAW264.7 cells

**FIGURE 7:**
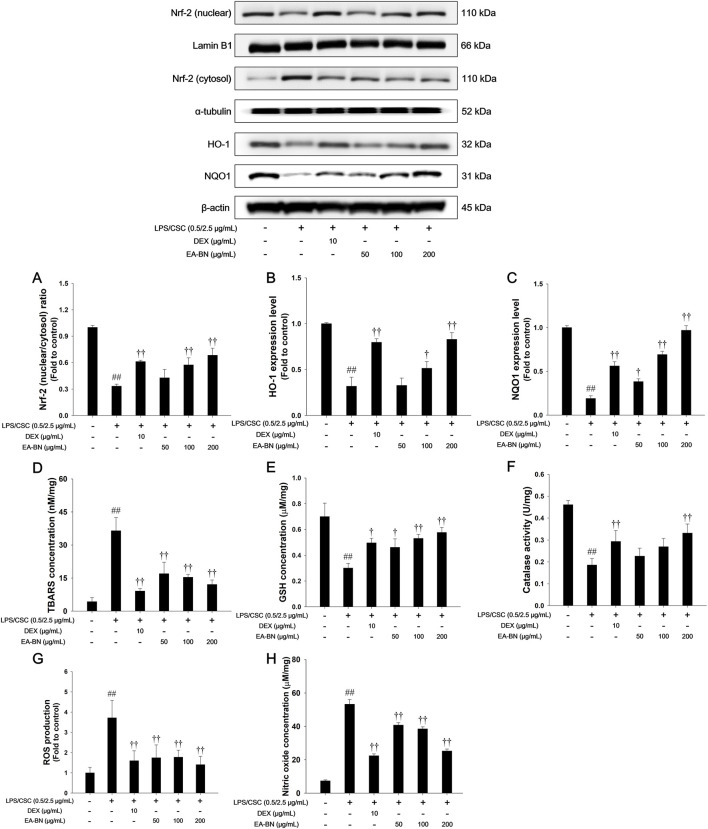
Effects of EA-BN on the Nrf-2/HO-1/NQO1 pathway and oxidative markers in LPS/CSC-stimulated RAW264.7 cells. Protein expression of **(A)** Nrf-2, **(B)** HO-1, and **(C)** NQO1 was examined by Western blot, with Lamin B1, α-tubulin, and β-actin used as loading controls. Oxidative stress parameters were evaluated, including **(D)** TBARS levels, **(E)** GSH content, **(F)** catalase activity, **(G)** ROS production, and **(H)** NO concentration. Cells were cultured in DMEM containing 0.1% FBS, pretreated with EA-BN (50, 100, and 200 μg/mL) for 1 h, and subsequently exposed to LPS/CSC (0.5/2.5 μg/mL) for either 2 h (for Nrf-2, HO-1, NQO1, TBARS, GSH, and catalase) or 24 h (for ROS and NO measurement). Con: Non-stimulated cells; LPS/CSC: LPS/CSC-stimulated RAW264.7 cells; DEX: dexamethasone (10 μg/mL) + LPS/CSC-stimulated RAW264.7 cells; EA-BN: EA-BN (50, 100 and 200 μg/mL) + LPS/CSC-stimulated RAW264.7 cells. The values are expressed as the means ± SD (*n* = 3). ^##^ *P* < 0.01, significantly different from control; ^††^ *P* < 0.01, significantly different from LPS/CSC-stimulated RAW264.7 cells.

As shown in Figure 7, stimulation with LPS/CSC led to a marked reduction in Nrf-2 nuclear translocation and significantly downregulated the expression of HO-1 and NQO1 in RAW264.7 cells. In contrast, treatment with EA-BN effectively promoted Nrf-2 translocation into the nucleus and upregulated the expression of its downstream antioxidant enzymes, HO-1 and NQO1 (Figures 7A–C). Furthermore, EA-BN administration resulted in a significant decrease in oxidative stress markers, including TBARS, ROS, and NO, while also leading to a notable increase in catalase activity (Figures 7D–H).

### 3.9 Effects of EA-BN on TXNIP/NLRP3 inflammasome and apoptotic signaling pathway in LPS/CSC-stimulated RAW264.7 cells

**FIGURE 8:**
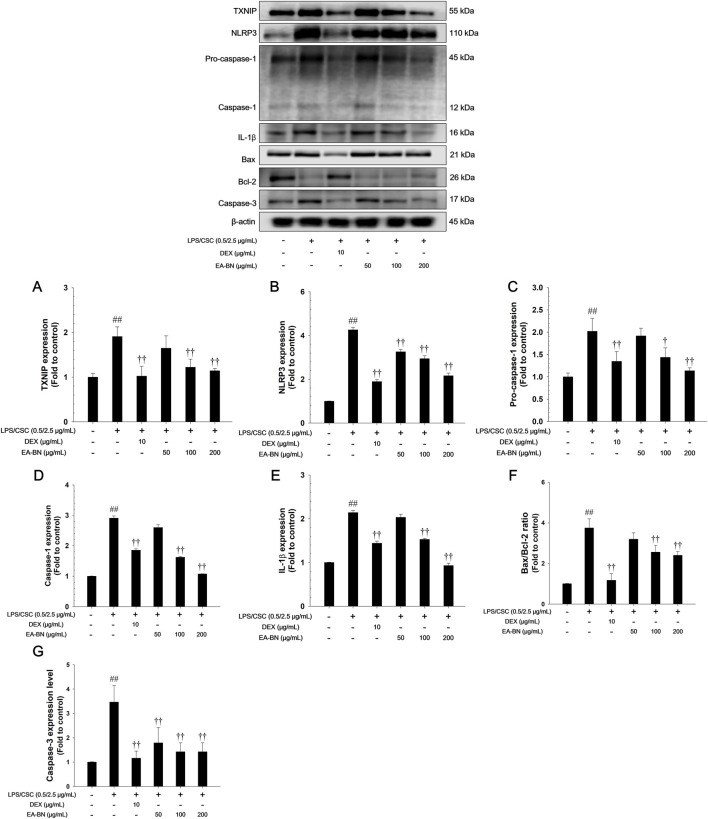
Effects of EA-BN on TXNIP/NLRP3 inflammasome and Bax/Bcl-2/caspase-3 signaling pathway in LPS/CSC-stimulated RAW264.7 cells. The expression of **(A)** TXNIP, **(B)** NLRP3, **(C)** pro-caspase-1, **(D)** caspase-1, **(E)** IL-1β, **(F)** Bax/Bcl-2 ratio, **(G)** caspase-3 were analyzed by western blot (β-actin: loading control). The culture medium was changed DMEM (0.1% FBS) and treated with EA-BN (25, 50 and 100 μg/mL) for 1 h and incubated with LPS (0.5 μg/mL) for 30 min (NLRP3), 6 h (TXNIP, pro-caspase-1, and caspase-1), and 24 h (IL-1β, Bax, Bcl-2, and caspase-3), respectively. Con: Non-stimulated cells; LPS/CSC: LPS/CSC-stimulated RAW264.7 cells; DEX: dexamethasone (10 μg/mL) + LPS/CSC-stimulated RAW264.7 cells; EA-BN: EA-BN (50, 100 and 200 μg/mL) + LPS/CSC-stimulated RAW264.7 cells. The values are expressed as the means ± SD (*n* = 3). ^##^ *P* < 0.01, significantly different from control; ^††^ *P* < 0.01, significantly different from LPS/CSC-stimulated RAW264.7 cells.

The LPS/CSC-stimulated RAW264.7 cells exhibited a notable increase in TXNIP and NLRP3 expression, and also showed elevated levels of the active forms of caspase-1 and IL-1β, compared to non-stimulated controls (Figures 8A–E). In contrast, EA-BN treatment significantly reduced the expression of TXNIP and NLRP3, and effectively inhibited the activation of caspase-1 and IL-1β. In addition, LPS/CSC exposure led to a notable increase in the Bax/Bcl-2 ratio, reflecting a shift toward pro-apoptotic signaling and the activation of caspase-3 in lung cells. Conversely, EA-BN treatment significantly lowered the Bax/Bcl-2 ratio and suppressed caspase-3 cleavage (Figures 8F,G).

### 3.10 Effects of TXNIP-specific siRNA on TXNIP/NLRP3 inflammasome and apoptotic signaling pathway in LPS/CSC-stimulated RAW264.7 cells

**FIGURE 9:**
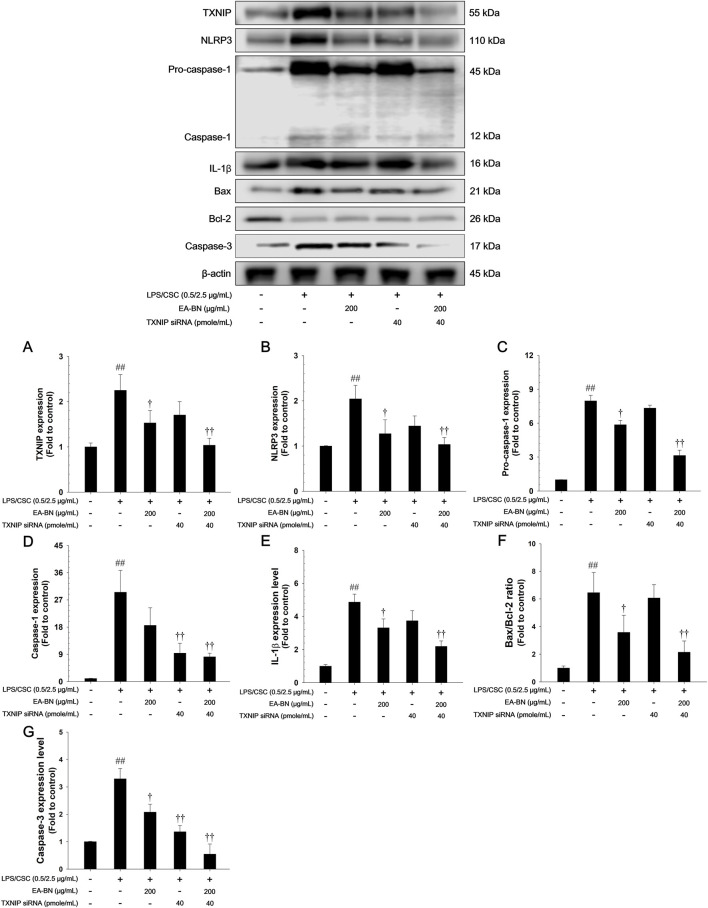
Effect of TXNIP knockdown and EA-BN on the TXNIP/NLRP3 inflammasome and Bax/Bcl-2/caspase-3 signaling pathway in LPS/CSC-stimulated RAW264.7 cells. Western blot analysis was performed to evaluate **(A)** TXNIP, **(B)** NLRP3, **(C)** pro-caspase-1, **(D)** caspase-1, **(E)** IL-1β, **(F)** Bax/Bcl-2 ratio, **(G)** caspase-3 expression, with β-actin serving as a loading control. Cells were maintained in DMEM (0.1% FBS), transfected with TXNIP-specific siRNA or treated with EA-BN (200 μg/mL) for 1 h, followed by stimulation with LPS/CSC (0.5/2.5 μg/mL) for 30 min (NLRP3), 6 h (TXNIP, pro-caspase-1, caspase-1), and 24 h (IL-1β, Bax, Bcl-2, and caspase-3). Con: Non-stimulated cells; **(A)** TXNIP, **(B)** NLRP3, **(C)** pro-caspase-1, **(D)** caspase-1, **(E)** IL-1β, **(F)** Bax/Bcl-2 ratio, **(G)** caspase-3LPS/CSC: LPS/CSC-stimulated RAW264.7 cells; EA-BN: EA-BN (200 μg/mL) + LPS/CSC-stimulated RAW264.7 cells; TXNIP siRNA: TXNIP siRNA (40 pmole/mL) + LPS/CSC-stimulated RAW264.7 cells; EA-BN + TXNIP siRNA: EA-BN (200 μg/mL) + TXNIP siRNA (40 pmole/mL) + LPS/CSC-stimulated RAW264.7 cells. The values are expressed as the means ± SD (*n* = 3). ^##^ *P* < 0.01, significantly different from control; ^††^ *P* < 0.01, significantly different from LPS/CSC-stimulated RAW264.7 cells.

To investigate the role of TXNIP in LPS/CSC-induced inflammatory and apoptotic responses, TXNIP-specific siRNA was transfected into RAW264.7 cells, followed by stimulation with LPS/CSC (0.5/2.5 μg/mL). As shown in Figures 9A–E, TXNIP knockdown significantly suppressed the expression of NLRP3 and reduced the active forms of caspase-1 and IL-1β, compared to the LPS/CSC-only group. In addition, TXNIP-specific siRNA transfection markedly lowered the Bax/Bcl-2 ratio and inhibited caspase-3 cleavage, indicating that apoptotic signaling was effectively attenuated (Figures 9F,G). These results demonstrate that TXNIP silencing mitigates both inflammasome activation and apoptosis, further supporting the anti-inflammatory and anti-apoptotic mechanism of EA-BN observed in earlier experiments.

### 3.11 EA-BN inhibited genes related to metabolic pathways from LPS/CSC-induced COPD model

**FIGURE 10:**
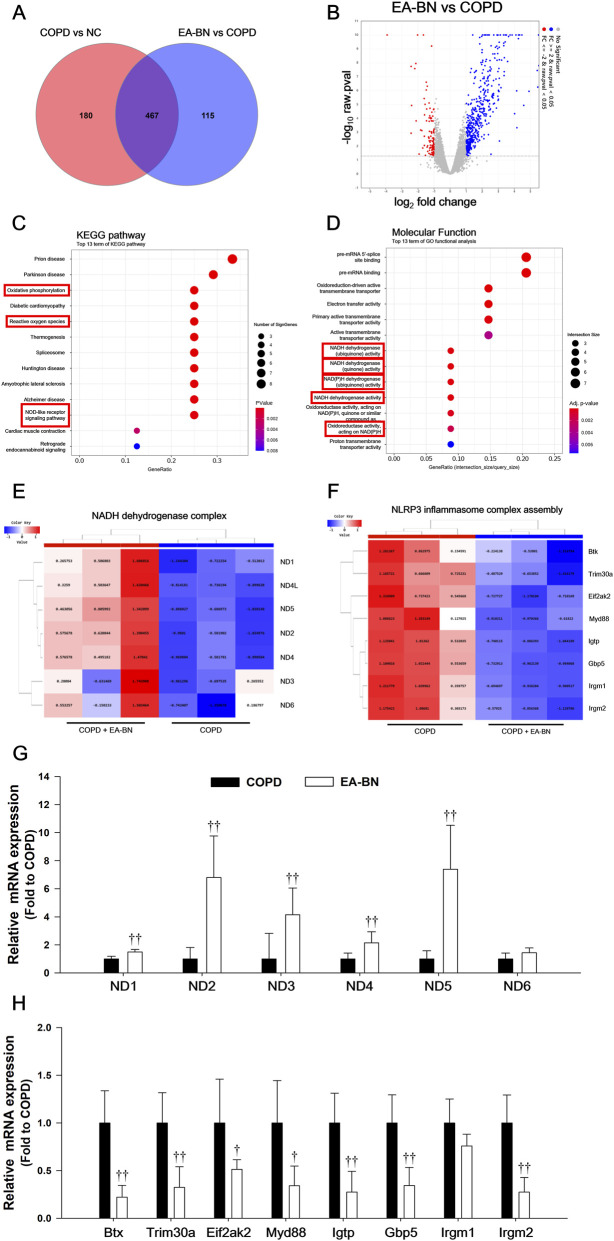
Transcriptomic analysis of EA-BN mechanisms in LPS/CSC-induced COPD. **(A)** Venn diagram illustrating overlapping genes and differentially expressed genes (DEGs) between COPD vs. NC and EA-BN vs. COPD lung tissues. **(B)** Volcano plot depicting DEGs in the EA-BN vs. COPD comparison. **(C)** KEGG enrichment analysis. **(D)** molecular function annotation of the key targets. **(E)** and **(F)** Heatmap representation of transcriptomic DEGs. **(G)** and **(H)** Real-time PCR analysis further validated the transcriptomic results, confirming the differential gene expression patterns observed in RNA-seq analysis. COPD: LPS/CSC-induced mice; EA-BN: EA-BN (200 mg/kg) + COPD. The values are expressed as the means ± SD (*n* = 7/group). ^†,††^ *P* < 0.05, *P* < 0.01, significantly different from COPD.

To elucidate the mechanisms underlying the preventive action of EA-BN in COPD, RNA-seq analysis of lung tissues was conducted. Venn diagram comparison revealed 647 differentially expressed genes (DEGs) between COPD and normal control (NC) mice, and 582 DEGs between EA-BN-treated and COPD mice, with 467 genes overlapping between the two groups (Figure 10A). As shown in Figure 10B, EA-BN treatment upregulated 473 genes and downregulated 109 genes. KEGG enrichment analysis of these DEGs indicated significant involvement of oxidative phosphorylation, reactive oxygen species, and NOD-like receptor signaling pathways (Figure 10C). Consistently, molecular function analysis highlighted enrichment in NADH dehydrogenase activity (Figure 10D). Heatmap visualization further demonstrated that EA-BN markedly increased the expression of mitochondrial NADH dehydrogenase complex genes, including ND1-ND6, while also modulating genes associated with the NLRP3 inflammasome complex assembly, such as Btk, Trim30a, Eif2ak2, Myd88, Igtp, Gbp5, Irgm1, and Irgm2 (Figures 10E,F). Heatmap analysis revealed that EA-BN upregulated mitochondrial NADH dehydrogenase complex genes (ND1-ND6) while downregulating transcripts associated with NLRP3 inflammasome assembly, such as Btk, Trim30a, Eif2ak2, Myd88, Igtp, Gbp5, Irgm1, and Irgm2 (Figures 10E,F). These transcriptomic trends were confirmed by real-time PCR analysis of lung tissues from LPS/CSC-exposed mice, showing increased expression of ND1-ND6 and decreased expression of inflammasome-related genes (Figures 10G,H). Collectively, these findings indicate that EA-BN alleviates COPD by enhancing mitochondrial oxidative phosphorylation and redox homeostasis while suppressing NLRP3-mediated inflammatory signaling.

## 4 Discussion

COPD is a progressive and treatment-resistant pulmonary disorder that currently represents the third most common cause of chronic disease and mortality worldwide. Its global prevalence is anticipated to reach 600 million by 2050 (Boers et al., 2023). To improve associated preventive options, plant-derived compounds have attracted increasing attention as potential alternatives or complementary preventive agents for COPD management (Kokkinis et al., 2024; Sami et al., 2021). In this study, we evaluated the protective effects of EA-BN in LPS/CSC-stimulated RAW264.7 cells and a COPD animal model established through exposure to LPS/CSC. EA-BN treatment markedly suppressed Th-1 cytokine production (IL-6, IL-1β, TNF-α) and diminished inflammatory cell infiltration in BALF. Lung function assessments indicated that EA-BN improved pulmonary mechanics by restoring total and tissue elastance, lowering airway resistance, and increasing the FEV_0.1_/FVC ratio. Histological examination revealed decreased peribronchial inflammation and preservation of alveolar structure. Furthermore, EA-BN showed antioxidant activity, reducing ROS, NO, and TBARS levels while enhancing catalase activity through activation of the Nrf-2/HO-1/NQO1 pathway. In addition, EA-BN inhibited TXNIP/NLRP3 inflammasome activation, as shown by reduced caspase-1 and IL-1β expression, and mitigated apoptosis by lowering the Bax/Bcl-2 ratio and cleaved caspase-3 levels in both RAW264.7 cells and the COPD model.

Chronic exposure to cigarette smoke contains numerous toxic compounds, particularly potent oxidants, that drive the excessive production of ROS (Pinto et al., 2017). Elevated ROS levels disrupt the oxidant-antioxidant balance, resulting in airway epithelial cell injury and alveolar destruction, which are key pathological features of COPD (Cipollina et al., 2022; Deng et al., 2023). Nrf-2 functions as a key regulator of redox homeostasis in COPD by relieving Keap-1 inhibition under oxidative stress, translocating to the nucleus, and inducing antioxidant gene expression. Its activation upregulates detoxifying enzymes (NQO1 and HO-1), enhances GSH synthesis and catalase activity, and reduces MDA accumulation, thereby mitigating ROS-induced cellular injury and maintaining redox stability (Cha et al., 2023; Qi and Fei, 2025; Zhang et al., 2024). In the present study, EA-BN administration significantly enhanced Nrf-2/HO-1/NQO1 expression while reducing ROS, NO, and TBARS levels and simultaneously restored GSH content and catalase activity in LPS/CSC-induced mice. Consistently, RNA-seq analysis revealed that EA-BN upregulated genes encoding components of the NADH dehydrogenase complex (ND1-6) in the lung tissues of mice exposed to LPS/CSC. Nrf-2/HO-1 activation has been reported to alleviate oxidative stress by enhancing GSH levels and catalase activity, in turn reducing pulmonary inflammation in LPS- and cigarette smoke-induced COPD models (Qi and Fei, 2025; Zhou et al., 2024). These findings suggest that the protective effects of EA-BN in the COPD model used herein are strongly linked to the suppression of oxidative stress through the activation of the Nrf-2/HO-1/NQO1 pathway and its associated antioxidant activities.

Elevated ROS levels in COPD activate the redox-sensitive protein TXNIP, which promotes NLRP3 inflammasome assembly with maturation of IL-1β and IL-18 (Kim W et al., 2024; Zhou et al., 2010). Previous studies have demonstrated that cigarette smoke and LPS exposure activate purinergic and toll-like receptor pathways, leading to P2X7R-mediated NLRP3 inflammasome activation, NF-κB-dependent cytokine production, and caspase-3-associated apoptosis in lung tissue (Dou et al., 2025; Lucattelli et al., 2011; Ozkanlar et al., 2025). These pathways represent an oxidative stress-driven inflammatory-apoptotic axis in COPD pathogenesis. These cytokines further recruit macrophages and neutrophils, which release Th1 cytokines, ROS, and proteolytic enzymes, thereby amplifying airway inflammation (Kotlyarov, 2022; Bezerra et al., 2023; Wu et al., 2022). In addition to its pro-inflammatory role, TXNIP initiates mitochondrial apoptotic signaling, which disrupts the Bax/Bcl-2 balance. It causes mitochondrial membrane permeabilization, cytochrome c release, and caspase-3 activation, ultimately leading to apoptosis of alveolar epithelial cells (Kim J et al., 2024). TXNIP-driven inflammation and apoptosis accelerate extracellular matrix degradation and alveolar destruction (Liu et al., 2023; Hua et al., 2025). These alterations are reflected in impaired lung function, as evidenced by reduced elastance and decreased FEV/FVC ratios (Soehnlein et al., 2017; Qi and Fei, 2025; Vogelmeier et al., 2017). In our study, EA-BN administration significantly inhibited Th1 cytokine production, inflammatory cell infiltration, and alveolar destruction in the lung tissues. Consequently, it contributed to the improvement of pulmonary function, as evidenced by the recovery of elastance parameters and FEV_0.1_/FVC ratio. EA-BN suppressed TXNIP/NLRP3 inflammasome activation and downregulated caspase-1 and IL-1β expression in both LPS/CSC-exposed mice and RAW264.7 cells. EA-BN attenuated apoptosis by reducing TXNIP expression, lowering the Bax/Bcl-2 ratio, and inhibiting caspase-3 activation. Combined treatment with EA-BN and TXNIP-specific siRNA showed an even stronger effect *in vitro*. RNA-seq analysis further revealed that EA-BN downregulated multiple genes involved in NLRP3 inflammasome assembly (Btk, Trim30a, Eif2ak2, Myd88, Igtp, Gbp5, Irgm1, and Irgm2) in the lung tissues. Consistent with previous findings, pharmacological inhibition of TXNIP/NLRP3 and Bax/Bcl-2/caspase-3 signaling has been shown to alleviate inflammation and apoptosis in COPD models induced by exposure to cigarette smoke (Hua et al., 2025; Mahalanobish et al., 2020; Wang et al., 2025). Our results indicated that EA-BN exerts protective effects against COPD by targeting TXNIP-mediated inflammatory and apoptotic pathways *in vivo* and *in vitro*.

*Boehmeria nivea* (L.) Gaud. has long been recognized in traditional Korean medicine for its diverse pharmacological properties, including anti-inflammatory, hepatoprotective, diuretic, and fever-reducing activities (Bae, 2000; Lin et al., 1998). Experimental research has further demonstrated that BN suppresses inflammatory signaling by inhibiting MAPK phosphorylation in LPS-activated RAW264.7 macrophages (Sung et al., 2013). Moreover, phytochemical analyses have identified several bioactive constituents of EA-BN, including rutin, epicatechin, luteolin-7-glucoside, and caffeic acid (Lee et al., 2020; Sung et al., 2013). In the present study, we emphasized the role of major components, ethyl gallate, caffeic acid, epicatechin, α-linolenic acid and pheophorbide A. Caffeic acid and ethyl gallate have been shown to alleviate oxidative stress by activating the Nrf-2 signaling pathway, thereby enhancing antioxidant defense mechanisms and reducing ROS production in LPS-exposed mice (Mehla et al., 2013; Yu et al., 2024). Pheophorbide A exhibited marked anti-inflammatory activity by suppressing nitric oxide production in LPS-stimulated RAW264.7 cells (Islam et al., 2013). Furthermore, epicatechin and α-linolenic acid were reported to attenuate inflammatory responses through the inhibition of NLRP3 inflammasome activation in cigarette smoke-exposed rats and LPS-stimulated RAW264.7 cells (Liu et al., 2022; Tian et al., 2021). In addition, *B. nivea* has been shown to exhibit excellent *in vivo* safety. Oral administration of BN leaf extract at doses up to 2 g/kg/day for 28 days produced no signs of systemic or organ toxicity in rats, as evidenced by normal hematological, biochemical, and histopathological profiles (Mu et al., 2020). Similarly, a single oral dose of up to 32 g/kg in pregnant mice caused no maternal or fetal toxicity (Tian et al., 2011). These results demonstrate the high tolerability of BN, supporting its suitability for further pharmacological and preventive evaluation. Collectively, these observations suggest that EA-BN has preventive significance in COPD, a disorder driven by chronic oxidative stress and airway inflammation. However, the specific molecular actions of its active metabolites in COPD models induced by LPS/CSC exposure remain insufficiently characterized, indicating the need for further mechanistic studies.

This study has several limitations. Although the LPS/CSC-induced COPD model does not fully replicate the prolonged exposure and chronic structural remodeling observed in cigarette smoke-induced models, it effectively reflects the principal pathological characteristics of COPD, including acute airway inflammation, increased pro-inflammatory cytokine levels, and impaired pulmonary function, as indicated by decreased FEV_0.1_/FVC ratios and altered elastance. Nevertheless, despite its relatively short duration, this model serves as a reliable and reproducible model for examining the interplay between oxidative stress and inflammation, which are key drivers of COPD pathogenesis, and for assessing the preventive potential of antioxidant and anti-inflammatory agents. Additionally, quantitative phytochemical profiling was not conducted, which limits the ability to determine the predominant bioactive constituents of EA-BN. Further study will include detailed quantitative analyses to clarify the contribution of individual compounds to its biological activity.

## 5 Conclusion

To the best of our knowledge, this is the first study to demonstrate that EA-BN effectively mitigates ROS-driven oxidative stress, airway inflammation, and apoptotic responses in both an LPS/CSC-induced COPD mouse model and LPS/CSC-stimulated RAW264.7 macrophages. EA-BN treatment significantly reduced Th1 cytokine levels (IL-6, IL-1β, and TNF-α), inflammatory cell infiltration, and alveolar destruction, leading to improvements in lung function, as evidenced by restoration of total and tissue elastance and the FEV_0.1_/FVC ratio. At the molecular level, these protective effects were mediated by the activation of the Nrf2/HO-1/NQO1 antioxidant defense pathway and the suppression of TXNIP-driven NLRP3 inflammasome signaling and Bax/Bcl-2/caspase-3-dependent apoptosis. Collectively, these findings provide compelling evidence supporting the preventive potential of EA-BN as a plant-derived therapeutic candidate for COPD management.

## Data Availability

The original contributions presented in the study are publicly available. This data can be found here: NCBI Gene Expression Omnibus (GEO; https://www.ncbi.nlm.nih.gov/geo/), accession number GSE315863.
